# Inhibition of Orai1‐mediated Ca^2+^ entry enhances chemosensitivity of HepG2 hepatocarcinoma cells to 5‐fluorouracil

**DOI:** 10.1111/jcmm.13029

**Published:** 2016-11-23

**Authors:** Bao‐Dong Tang, Xin Xia, Xiao‐Fei Lv, Bei‐Xin Yu, Jia‐Ni Yuan, Xiao‐Yi Mai, Jin‐Yan Shang, Jia‐Guo Zhou, Si‐Jia Liang, Rui‐Ping Pang

**Affiliations:** ^1^ Department of GastroenterologyThe First Affiliated HospitalSun Yat‐Sen UniversityGuangzhouChina; ^2^ Department of Thoracic SurgeryThe First Affiliated HospitalSun Yat‐Sen UniversityGuangzhouChina; ^3^ Department of Pharmacology, Cardiac and Cerebrovascular Research CenterZhongshan School of MedicineSun Yat‐Sen UniversityGuangzhouChina; ^4^ Guangdong Province Key Laboratory of Brain Function and DiseaseZhongshan School of MedicineSun Yat‐Sen UniversityGuangzhouChina; ^5^ Department of PhysiologyPain Research CenterZhongshan School of MedicineSun Yat‐Sen UniversityGuangzhouChina

**Keywords:** 5‐fluorouracil, chemosensitivity, autophagy, store‐operated Ca^2+^ entry, Orai1

## Abstract

Increasing evidence supports that activation of store‐operated Ca^2+^ entry (SOCE) is implicated in the chemoresistance of cancer cells subjected to chemotherapy. However, the molecular mechanisms underlying chemoresistance are not well understood. In this study, we aim to investigate whether 5‐FU induces hepatocarcinoma cell death through regulating Ca^2+^‐dependent autophagy. [Ca^2+^]_i_ was measured using fura2/AM dye. Protein expression was determined by Western blotting and immunohistochemistry. We found that 5‐fluorouracil (5‐FU) induced autophagic cell death in HepG2 hepatocarcinoma cells by inhibiting PI3K/AKT/mTOR pathway. Orai1 expression was obviously elevated in hepatocarcinoma tissues. 5‐FU treatment decreased SOCE and Orai1 expressions, but had no effects on Stim1 and TRPC1 expressions. Knockdown of Orai1 or pharmacological inhibition of SOCE enhanced 5‐FU‐induced inhibition of PI3K/AKT/mTOR pathway and potentiated 5‐FU‐activated autophagic cell death. On the contrary, ectopic overexpression of Orai1 antagonizes 5‐FU‐induced autophagy and cell death. Our findings provide convincing evidence to show that Orai1 expression is increased in hepatocarcinoma tissues. 5‐FU can induce autophagic cell death in HepG2 hepatocarcinoma cells through inhibition of SOCE *via* decreasing Orai1 expression. These findings suggest that Orai1 expression is a predictor of 5‐FU sensitivity for hepatocarcinoma treatment and blockade of Orai1‐mediated Ca^2+^ entry may be a promising strategy to sensitize hepatocarcinoma cells to 5‐FU treatment.

## Introduction

Hepatocellular carcinoma (HCC) is one of the most common malignant tumours and the third leading cause of cancer‐induced death worldwide, behind only lung and gastric cancers 1. It accounts for 695,900 deaths per year, and half of these deaths are estimated to occur in China 2. Clinically, despite the improvement in surgical excision and liver transplantation, most HCC patients are not suitable for surgical resection due to extensive disease or poor liver function 1. Therefore, the systemic chemotherapy may be the most important therapeutic strategy for HCC.

5‐FU is the most commonly used chemotherapeutic drug for the treatment of a variety of tumour by induction of cancer cells apoptosis 3, 4. As reported in many solid tumours, most patients exert high chemosensitivity to 5‐FU at the beginning of therapy. Unfortunately, cancer cells can quickly acquire chemoresistance, resulting in very low response to systemic chemotherapy and serious side effects 3. Thus, it is urgent to develop novel promising strategies that can enhance chemotherapeutic efficacy in cancer treatments.

Store‐operated Ca^2+^ channels (SOCs) represent the major Ca^2+^‐entry pathway in non‐excitable cells, which is activated by triphosphate (IP3)‐mediated depletion of endoplasmic reticulum (ER) Ca^2+^ in response to the activation of phospholipase C‐coupled receptors 5. Moreover, SOCE has been confirmed as the main Ca^2+^ influx pathway regulating Ca^2+^ homeostasis and cell survival of human hepatoma cell lines 6, 7. Stromal interaction molecule 1 (STIM1) embedded in the ER membrane, and the calcium release‐activated calcium channel protein 1 (CRACM1, also called Orai1) and transient receptor potential canonica l (TRPC1) located in the plasma membrane are the potential molecular components of SOCs 8, 9, 10. Upon ER store depletion, STIM1 aggregates and translocates to plasma membrane and then interacts directly with Orai1 and/or TRPC1 to form open SOCs that cause SOCE 5, 8. Interestingly, the three constituents of SOCE, Stim1, Orai1 and TRPC1, have been detected in liver cells and associated with the initiation and progression of liver cancer 6, 7, 11, 12. Previous work in HCC‐LM3 hepatoma cells showed that blockade of STIM‐mediated SOCE decreased the focal adhesion turnover and thus inhibited cell migration 6. Furthermore, silencing of TRPC1 was associated with inhibition of hepatocarcinoma cell proliferation by causing cell cycle arrest 7, 12. These results suggest SOCs play a critical role in regulating chemoresistance of hepatocarcinoma cells.

Autophagy is a catabolic process responsible for the delivery and degradation of proteins and organelles, which maintains cellular homeostasis during cellular stress situation 13, 14. Despite 5‐FU has been recently shown to induce autophagy in a number of human cancer cells 4, 15, 16, 17, the role of autophagy in 5‐FU‐mediated hepatocarcinoma cell death is still unclear. In this study, we investigated whether 5‐FU induces hepatocarcinoma cell death through regulating Ca^2+^‐dependent autophagy. Our results indicate that Orai1‐mediated SOCE contributes to the chemoresistance of hepatocarcinoma cells to 5‐FU‐induced autophagic cell death.

## Materials and methods

### Materials and reagents

5‐FU, haematoxylin, Hoechst 33258 dye, thapsigargin, 3‐methyladenine (3‐MA), chloroquine and SKF96365 were purchased from Sigma‐Aldrich (St. Louis., MO, USA). Dulbecco's modified Eagle's medium (DMEM), foetal bovine serum (FBS), Lipofectamine 2000, Opti‐MEM medium and Trizol reagent were obtained from Invitrogen (Carlsbad, CA, USA).

### Clinical research specimens

Paired liver tumour tissues and corresponding adjacent non‐tumour tissues from 20 HCC patients who underwent surgical resection between 2009 and 2012 were obtained from the 1st Affiliated Hospital, Sun Yat‐Sen University. Tissue specimens were quickly frozen after surgical resection and stored in liquid nitrogen. Histological evaluation of the tumour and non‐tumour tissues was performed by haematoxylin and eosin (H&E) staining, and each liver tumour specimen was matched with the adjacent non‐tumour tissue specimen from the same patient. This study was approved by the Medical Research Ethics Committee of Sun Yat‐sen University and conducted in accordance with the principles expressed in the Declaration of Helsinki. All patients signed an informed consent, and the data and samples were analysed anonymously.

### Cell culture

The human hepatocarcinoma cell line HepG2 was obtained from the cell line bank of the Chinese Academy of Sciences (Shanghai, China) and cultured in DMEM supplemented with 10% FBS, penicillin (100 U/ml) and streptomycin (100 mg/ml) at 37°C under a humidified atmosphere of 5% CO_2_ and 95% air.

### Cell viability assay

Cell viability was measured with Cell Counting Assay Kit‐8 (CCK‐8; Dojindo Molecular Technologies, MD, Japan), according to the manufacturer's instructions. HepG2 cells were seeded in 96‐well plates (1 × 10^5^ cells/well) followed by treatment of 5‐FU for 48 hrs. CCK‐8 reagent (10 μl/well) was added and incubated for 2 hrs. The absorbance was read at 450‐nm wavelength using a microplate reader (Bio‐Tek, VT, USA).

### Western blotting analysis

Western blotting was performed as we previously described 18, 19. Briefly, cells were washed twice with ice‐cold phosphate‐buffered saline and lysed with RIPA lysis buffer (Beyotime, Jiangsu, China) containing 1% protease inhibitor cocktail (Merck, CA, USA). To extract protein from the liver tissues, the snap‐frozen tissues were homogenized in lysis buffer supplemented with protease inhibitors. Cell or tissue debris was removed by centrifugation at 15,000 × *g* for 10 min. The protein content was quantified with BCA kit (Beyotime). Equal amount of protein was resolved on 8–12% SDS‐PAGE and transferred onto a polyvinylidene difluoride membrane (Millipore, Billerica, MA, USA). The membranes were probed with primary antibodies to LC3B‐I/II (1:1000 dilution), Beclin‐1 (1:1000 dilution), ATG5 (1:1000 dilution), p62/SQSTM1 (1:500 dilution), phospho‐AKT (1:500 dilution), AKT (1:1000 dilution), phospho‐mTOR (1:500 dilution), mTOR (1:1000 dilution), phospho‐p70S6K (1:1000 dilution), p70S6K (1:1000 dilution; Cell Signaling Technology, Billerica, MA, USA), Orai1 (1:1000 dilution; Alomone Labs, Jerusalem, Israel), TRPC1 (1:500 dilution; Santa Cruz Biotechnology, Santa Cruz, CA, USA), Stim1 (1:1000 dilution), phosphoserine (1:1000 dilution; Abcam, Cambridge, MA, USA) and β‐actin (1:1000 dilution; Beyotime). Appropriate secondary horseradish peroxidase‐conjugated antibodies (1:1000; Cell Signaling Technology) were used to label the proteins for 1 hr. Bands were visualized by enhanced chemiluminescence detection kit (Pierce, Thermo Scientific, Waltham, MA, USA) and quantified by IMAGEJ analysis software (NIH, Bethesda, MD, USA).

### Immunoprecipitation

Immunoprecipitation was performed as described previously 19, 20. Cell lysates were immunoprecipitated with Stim1 antibody overnight at 4°C, followed by incubation with Protein A/G–Sepharose (Santa Cruz Biotechnology) for 4 hrs. The immunoprecipitates were harvested by centrifugation at 2500 × *g* for 15 min. and washed three times with PBS. The protein was boiled in SDS loading buffer and subjected to Western blotting analysis using phosphoserine antibody.

### Plasmids transfection

GFP‐LC3 was a gift from Dr. Canzhao Liu (University of California, San Diego, CA, USA), and Orai1 plasmid was kindly provided by Dr. Weichiao Chang (Kaohsiung Medical University Hospital, Taiwan). The plasmid was diluted in Opti‐MEM medium without serum, and then, Lipofectamine 2000 was added to the diluted plasmid. The samples were kept at room temperature for 20 min. to form the transfection complexes. The complexes were added to the cells and were swirled gently to ensure uniform distribution. Six hours later, transfection complexes were removed and the cells were cultured in DMEM containing 10% FBS and antibiotics for 48 hrs.

### Analysis of autophagy by microscopy

Cells transfected with GFP‐LC3 were fixed in 4% paraformaldehyde for 30 min., and immunofluorescence was observed with a laser‐scanning confocal microscopy (FV500, Olympus, Shibuya‐ku, Tokyo, Japan). The nuclei were stained with Hoechst 33258. The average number of GPF‐LC3 puncta per GFP‐LC3 positive cell was assessed by counting 20 random fields of view (about 20 cells) per group in six independent experiments.

### Immunohistochemistry

Immunohistochemistry was performed using the streptavidin–biotin–peroxidase complex system as described previously 20, 21. Briefly, paraformaldehyde‐fixed, paraffin‐embedded sections (8 μm) cleared of paraffin in Citroclear and rehydrated through graded industrial methylated spirit series. After being blocked with 5% goat serum for 1 hr, the sections were incubated with Orai1 (1:100) antibody at 4°C overnight and then were treated with biotinylated secondary anti‐rabbit antibody (1:100, Vector Laboratories, Burlingame, CA, USA) for 30 min. at room temperature. The sections were incubated with streptavidin–biotin–peroxidase complex for 30 min. and visualized with DAB chromogen (Vector Laboratories), followed by counterstaining with haematoxylin.

### RNA extraction and quantitative real‐time PCR

Total RNA was extracted with the Trizol reagent according to the manufacturer's instructions. Two micrograms of total RNA was reverse‐transcribed using a PrimeScript RT reagent kit (Bio‐Rad Laboratories, Hercules, CA, USA). Quantitative real‐time PCR was performed using SYBR Green PCR master mix (Invitrogen) on a MyiQ Single Color Real‐time PCR Detection System (Bio‐Rad) for 32 cycles (95°C for 10 sec., 57°C for 1 min.) after an initial 3‐min incubation at 95°C. The fold change in expression of orai1 was calculated using the 2^−▵▵CT^ method with 18S rRNA as an internal control. The sequence‐specific primers (Sangon Biotech, Shanghai, China) were used as follows: Orai1, 5′‐GCCCTTCGGCCTGATCTTTA‐3′ (sense) and 5′‐TCCTGTAAGCGGGCAAACTC‐3′ (antisense); 18s rRNA5′‐CGGCTACCACATCCAAGGAA‐3′ (sense) and 5′‐CTGGAATTACCGCGGCT‐3′ (antisense).

### Intracellular Ca^2+^ ([Ca^2+^]_i_) measurement

[Ca^2+^]_i_ was measured as we previously described 18, 21. Briefly, cells were suspended in Ca^2+^‐free HBSS (130 mM NaCl, 4.8 mM KCl, 1.2 mM KH_2_PO_4_, 1.2 mM MgSO_4_, 25 mM NaHCO_3_, 10 mM glucose, 20 mM HEPES, 50 μM EGTA and 0.1% fatty acid free bovine serum albumin, pH 7.4) solution containing 2 μM fura2/AM for 45 min. at 37°C and treated with thapsigargin (Tg, 1 μM) to induce ER Ca^2+^ stores depletion before addition of 1 mM of CaCl_2_. Fluorescence emission was monitored at 510 nm using RF‐5301 fluorescence Spectrophotometer (Shimadzu, Tokyo, Japan) with an excitation at 340 and 380 nm. Fluorescence intensity was expressed as the ratio of fura‐2 fluorescence from excitation at 340 nm to that from excitation at 380 nm (F340/F380).

### RNA interference

The siRNA duplexes against Orai1 (Qiagen, Valencia, CA, USA) were transiently transfected with Hiperfect Transfection Reagent (Qiagen) according to the manufacturer's instructions as previously described 18, 21. The siRNA target sequence for Orai1 was 5′‐UUGCUCACCGCCUCGAUGUTG‐3′. A scrambled siRNA (Qiagen) was used as negative control. siRNA and Hiperfect Transfection Reagent were diluted in serum‐ and antibiotic‐free DMEM. The samples were incubated at room temperature for 10 min. to form the transfection complexes. The complexes were then added to the cells and were swirled gently to ensure uniform distribution. After incubation for 6 hrs at 37°C, transfection complexes were replaced with normal DMEM containing 10% FBS and antibiotics. Forty‐eight hours later, Western blot was used to examine the effect of Orai1 siRNA and scrambled siRNA on protein expression.

### Statistical analysis

All data were expressed as mean ± S.E.M. One‐way anova using the Bonferroni multiple comparison *post hoc* test with a 95% confidence interval or unpaired two‐tailed Student's *t*‐test was employed in SPSS 16.0 system (SPSS Inc., Chicago, IL, USA). *P* < 0.05 was considered to be statistically significant.

## Results

### 5‐FU induces cell death and autophagy in HepG2 cells

**Figure 1:**
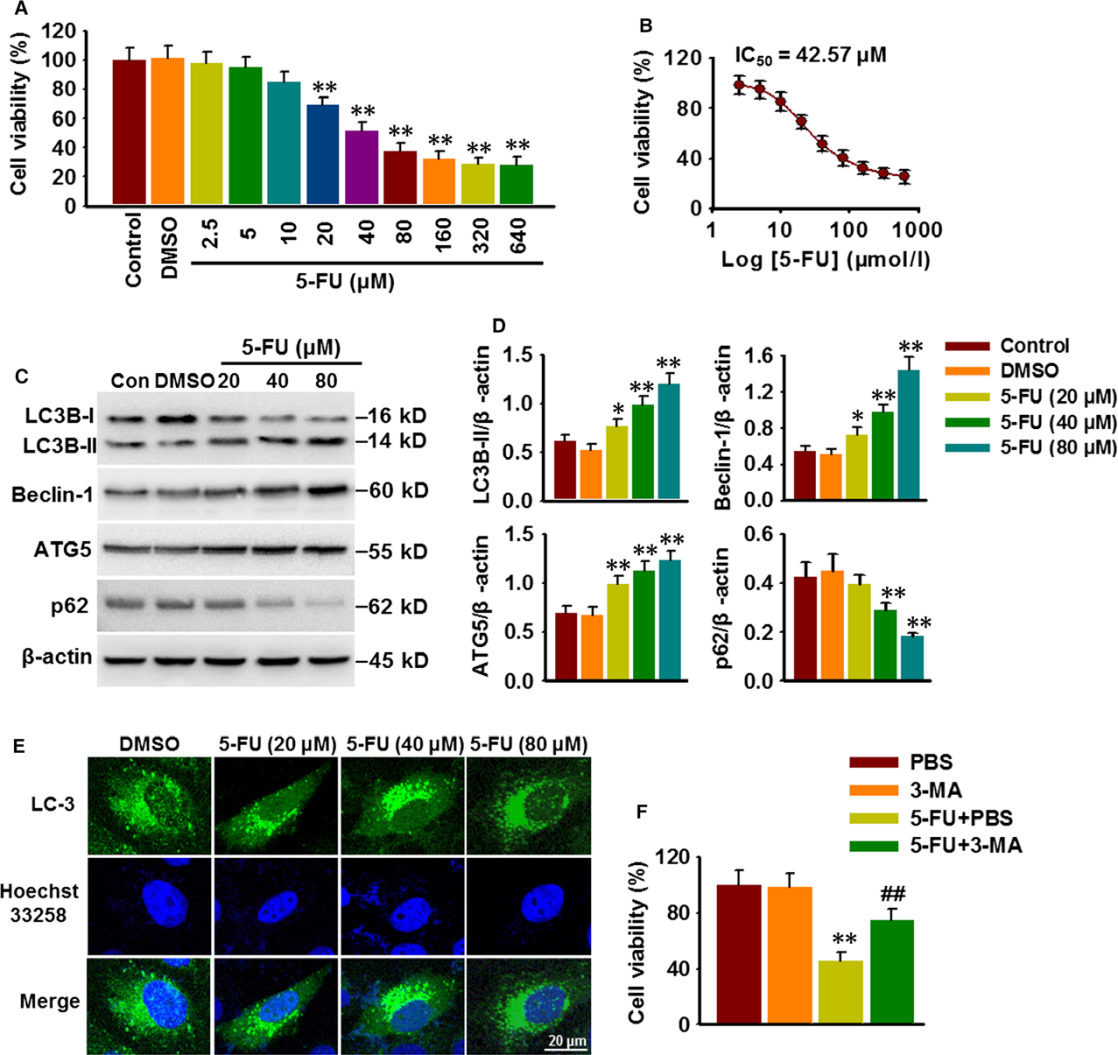
5‐FU induces cell death and autophagy in HepG2 cells. (**A**) Cells were cultured in the presence of varying concentrations of 5‐FU or DMSO for 48 hrs. HepG2 cell viability was analysed with CCK‐8 assay. *P < 0.05, **P < 0.01 *versus* DMSO,* n* = 6. (**B**) Dose–response curve for the effect of 5‐FU on cell viability. (**C**) The expression of autophagy‐associated markers LC3B, Beclin‐1, ATG5 and p62 was determined by Western blotting. (**D**) Bar diagram represents the densitometric analysis of these markers. *P < 0.05, **P < 0.01 *versus* DMSO,* n* = 4. (**E**) HepG2 cells transfected with GFP‐tagged LC‐3 were treated with different concentrations of 5‐FU for 48 hrs, and the formation of GFP‐LC3 puncta was observed using a laser‐scanning confocal microscopy. (**F**) Cells were pre‐treated with PBS or 3‐MA (5 μM) for 30 min. and then were treated with 5‐FU (80 μM) for another 48 hrs. HepG2 cell viability was examined by CCK‐8 assay. **P < 0.01 *versus* PBS, ##*P* < 0.01 *versus* 5‐FU+PBS,* n* = 6.

In consistent with previous studies 22, 23, 5‐FU treatment for 48 hrs decreased HepG2 cell viability in a concentration‐dependent manner (Fig. 1A). The IC_50_ value was 42.57 μM (Fig. 1B). To understand whether autophagy contributes to 5‐FU‐induced HepG2 cell death, protein expression of autophagy marker LC3B and lipidated LC3B‐II was determined. The results showed that 5‐FU (20–80 μM) treatment enhanced LC3B‐II level and reduced LC3B‐I expression in a concentration‐dependent manner, indicating increased transformation of LC3B‐I to LC3B‐II. Beclin‐1 and ATG5, another two autophagy‐associated proteins, were obviously up‐regulated after 5‐FU treatment. Correspondingly, the p62 protein, which is degraded in cells undergoing autophagy, was remarkably decreased after 5‐FU treatment (Fig. 1C and D). In addition, immunofluorescence analysis also showed increased number of the green punctate after 5‐FU treatment in GFP‐LC3 transfected HepG2 cells, which was consistent with the results of Western blotting (Fig. 1E). We next examined whether the altered autophagy is involved in the effect of 5‐FU on HepG2 cell death. Cell viability assay revealed that pre‐treatment with 3‐MA (5 μM), an autophagy inhibitor, remarkably attenuated 5‐FU‐induced cell death (Fig. 1F). These findings indicate that 5‐FU could induce autophagic cell death in HepG2 cells.

### Class I PI3K/AKT/mTOR pathway is required for 5‐FU‐activated autophagosome formation

**Figure 2:**
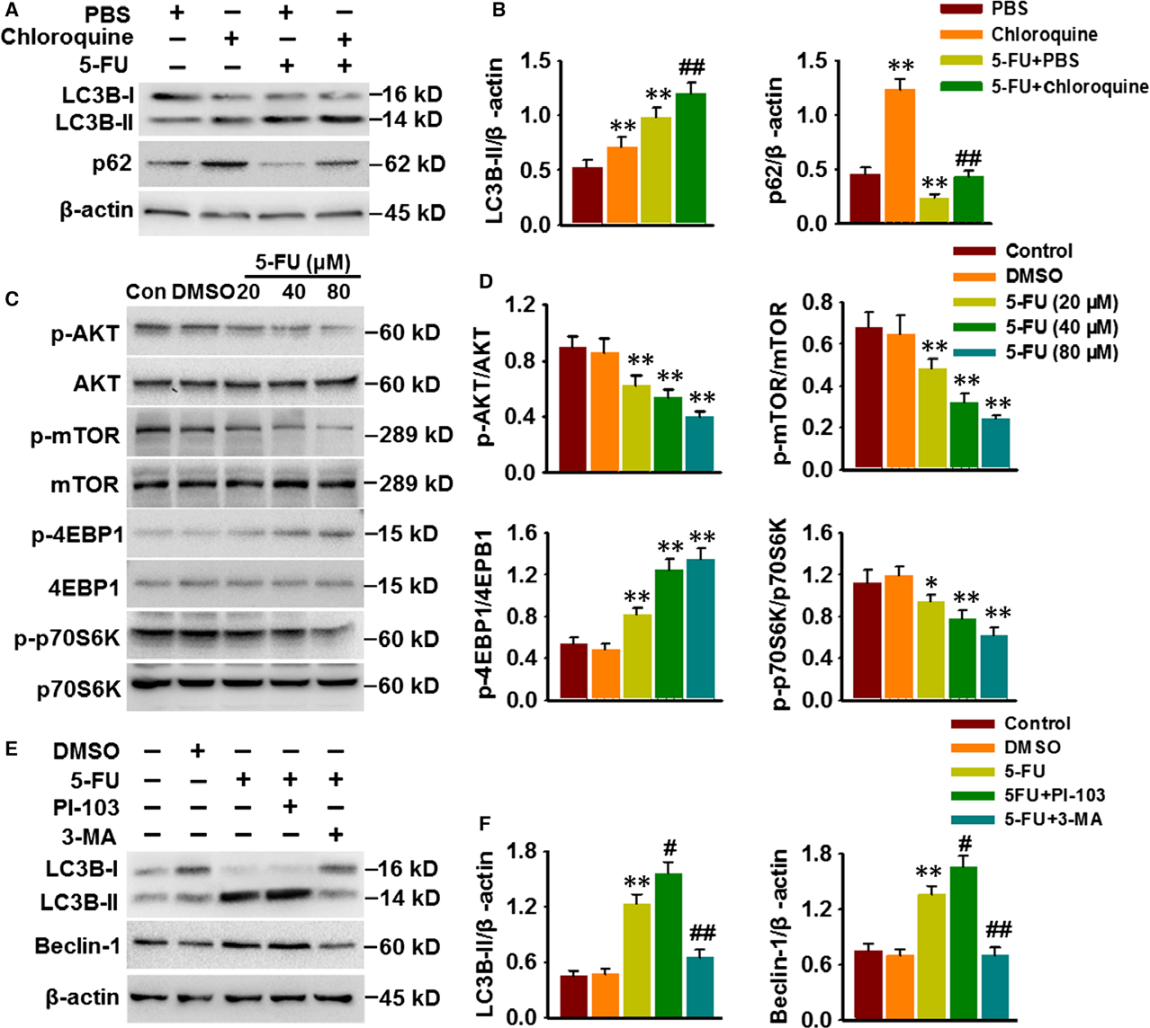
5‐FU induces autophagosome formation through PI3K/AKT/mTOR pathway. (**A**) HepG2 cells were pre‐treated with PBS or chloroquine (1 μM) for 30 min. and were then exposed 5‐FU (80 μM) for 48 hrs. LC3B and p62 expressions were detected by Western blotting. (**B**) Densitometric analysis of LC3B‐II and p62 protein expression. **P < 0.01 *versus* PBS, ##*P* < 0.01 *versus* 5‐FU+PBS,* n* = 5. (**C**) Western blotting images showing the phosphorylation of AKT, mTOR, 4EBP1 and p70S6K in HepG2 cells after 5‐FU treatment for 48 hrs. (**D**) Densitometric analysis of these protein expression was performed. *P < 0.05, **P < 0.01 *versus* DMSO,* n* = 6. (**E**) Cells were pre‐treated with PI‐103 (0.5 μM) or 3‐MA (5 μM) prior to incubation with 5‐FU (80 μM) for 48 hrs. Western blotting analysis of LC‐3B and Beclin‐1 expression. (**F**) Bar diagram represents the densitometric analysis of LC3B‐II and Beclin‐1. **P < 0.01 *versus* DMSO, #*P* < 0.05, ##*P* < 0.01 *versus* 5‐FU,* n* = 4.

To elucidate whether increased autophagosomes formation or decreased autophagosomes degradation contribute to 5‐FU‐induced autophagy, the effect of chloroquine (1 μM), a lysosomal inhibitor, on autophagy was examined. The results showed that chloroquine pre‐treatment further increased 5‐FU‐induced LC3B‐II formation and p62 accumulation (Fig. 2A and B), suggesting the activation of autophagosome formation rather than the inhibition of autophagosome degradation is the major mechanism underlying 5‐FU‐evoked autophagy.

PI3K/AKT/mTOR is one of the major signalling pathways involved in the regulation of autophagy 13. Western blotting showed that 5‐FU significantly decreased the phosphorylation of AKT and mTOR and subsequently inhibited the activation of p70S6K, an important kinase downstream target of mTOR. On the contrary, 4EBP1 phosphorylation was markedly increased in the cells treated with 5‐FU (Fig. 2C and D). As 3‐MA is also an inhibitor of class III PI3K, which was found to inhibit 5‐FU‐induced autophagic death, and AKT/mTOR pathway is predominantly activated by class I PI3K, we next used specific inhibitors for PI3K class I or III to further examine the role of PI3K on autophagy induced by 5‐FU. Pre‐treatment with PI‐103 (0.5 μM), an inhibitor of class I PI3K, enhanced 5‐FU‐induced LC3B‐II formation and Beclin‐1 expression, whereas 3‐MA treatment was associated with reduced protein expression of LC3B‐II and Beclin‐1 (Fig. 2E and F). Together, these results suggest that 5‐FU induces autophagy through inhibiting class I PI3K/AKT/mTOR signalling pathway.

### 5‐FU inhibits SOCE through down‐regulating Orai1 expression

**Figure 3:**
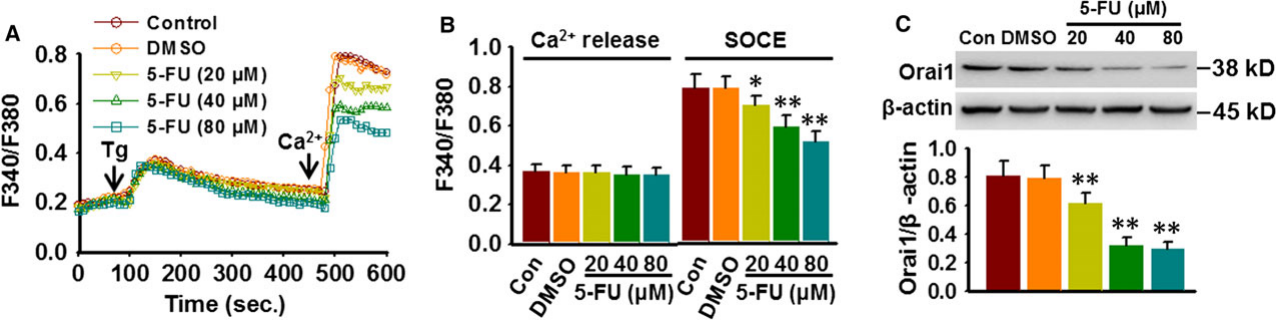
5‐FU inhibits SOCE through reducing Orai1 expression. (**A**) Cells were treated with various concentrations of 5‐FU for 48 hrs. Ca^2+^ images showing the thapsigargin (Tg, 1 μM)‐induced Ca^2+^ entry after addition of 1 mM Ca^2+^ in Ca^2+^‐free medium. (**B**) Bar diagram showing the fluorescence ratio (340/380) as mentioned in methods section. *P < 0.05, **P < 0.01 *versus* DMSO,* n* = 6. (**C**) HepG2 cells were treated with different 5‐FU concentrations for 48 hrs; Orai1 protein expression was examined by Western blotting. **P < 0.01 *versus* DMSO,* n* = 4.

[Ca^2+^]_i_ has been demonstrated to be important for autophagy and cancer cell survival 6, 13, 14. As displayed in Figure 3A, thapsigargin (Tg, 1 μM), a sarcoplasmic reticulum Ca^2+^ pump inhibitor, activated a transient Ca^2+^ release in the absence of extracellular Ca^2+^ and subsequently a pronounced Ca^2+^ entry after restoration of 1 mM Ca^2+^ in HepG2 cells. 5‐FU treatment for 48 hrs did not affect the resting [Ca^2+^]_i_ and Tg‐evoked Ca^2+^ release. However, in the presence of extracellular Ca^2+^, store‐mediated Ca^2+^ entry was significantly decreased after 5‐FU challenge (Fig. 3A and B). Moreover, Western blotting showed that 5‐FU concentration‐dependently decreased the expression of Orai1 protein, whereas Stim1 and TRPC1 expressions were unchanged (Fig. 3C and Fig. S1A and B). Although Stim1 has been recently reported to be phosphorylated following AKT activation 24, no changes in Stim1 phosphorylation were observed in DMSO‐treated and 5‐FU‐treated cells (Fig. S1C). These results suggest that reduced Orai1 expression might contribute to 5‐FU‐induced the loss of Ca^2+^ entry.

### Orai1 is overexpressed in hepatocarcinoma tissues and involved in SOCE in HepG2 cells

**Figure 4:**
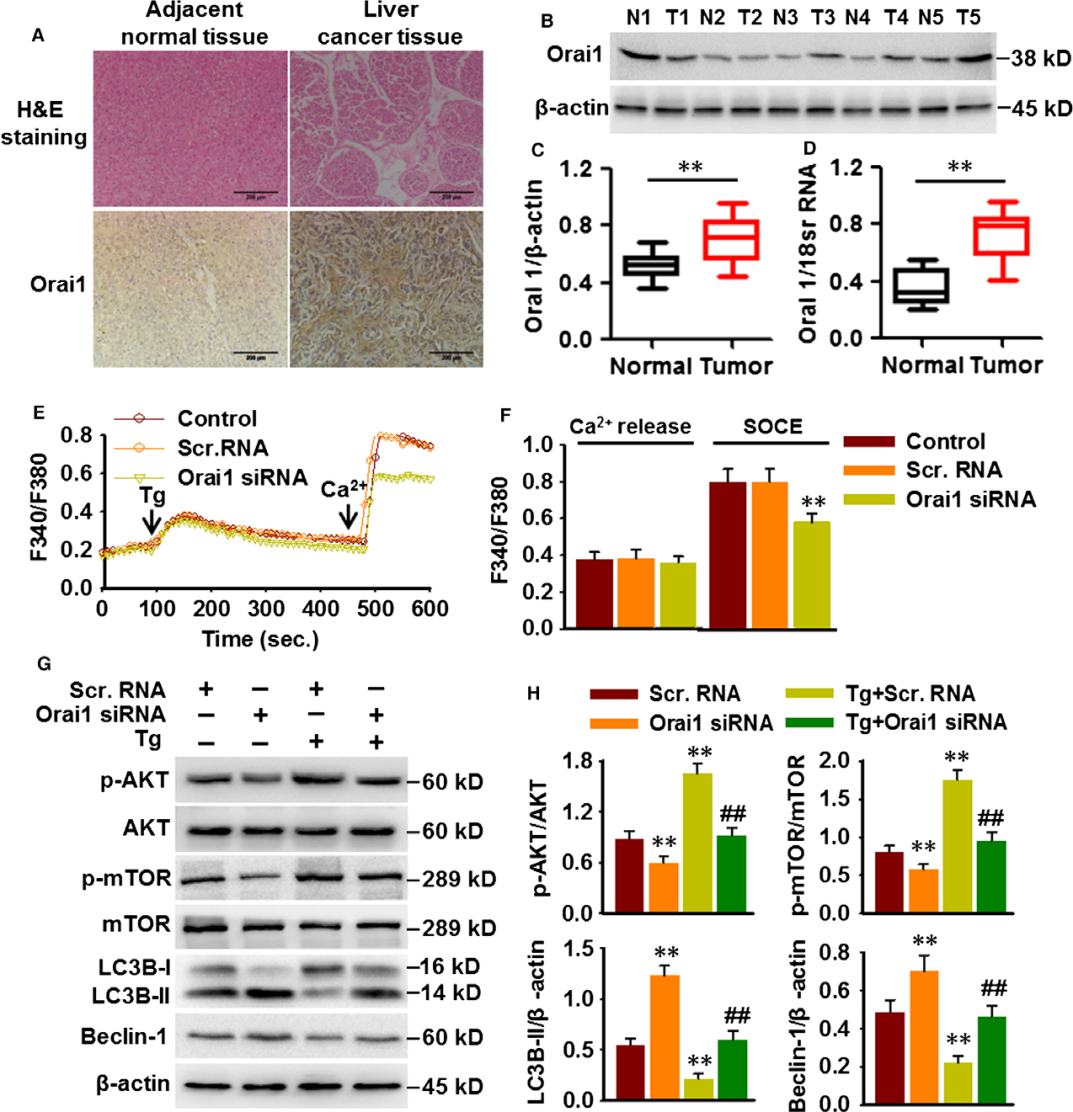
Orai1 is overexpressed in liver cancer tissues and involved in SOCE in HepG2 cells. (**A**) Histopathological changes in liver cancer and adjacent normal tissues from same hepatocarcinoma patients were examined by haematoxylin and eosin staining, and Orai1 expression was determined by immunohistochemistry. (**B**) Representative Western blotting analysis of Orai1 expression in liver cancer samples. (**C**) Densitometric analysis of Orai1 protein expression. (**D**) mRNA expression of Orai1 was analysed by quantitative real‐time PCR. **P < 0.01 *versus* normal tissue, *n* = 20. (**E**) HepG2 cells were transfected with Scr.RNA or Orai1 siRNA (50 nM) for 48 hrs. Ca^2+^ imaging was performed by using Fura2/AM probe, and analogue plots of the fluorescence ratio (340/380) were shown. (**F**) Quantification of fluorescence ratio (340/380). **P < 0.01 *versus* Scr.RNA,* n* = 4. (**G**) Cells were treated with Scr.RNA or Orai1 siRNA for 48 hrs, and then, thapsigargin (Tg, 1 μM) was added into the culture medium for another 20 min. The phosphorylation of AKT and mTOR, and the protein expression of LC3B and Beclin‐1 were determined by Western blotting. (**H**) Densitometric analysis of the above protein expression. **P < 0.01 *versus* Scr RNA, ##*P* < 0.01 *versus* Tg+Scr.RNA,* n* = 4.

Immunohistochemistry staining showed that Orai1 expression in tumour tissues was markedly increased compared with non‐tumour tissues (Fig. 4A). Western blotting data further demonstrated Orai1 protein expression was up‐regulated in 15 liver tumour samples, which accounted for 75% of the total 20 samples (Fig. 4B and C and Fig. S2). Consistently, Orai1 mRNA expression was also increased in liver tumour tissues compared to adjacent normal tissue (Fig. 4D), suggesting that the changes in Orai1 expression may be associated with the biological characteristic of normal cells and cancer cells. We next examined whether Orai1 is involved in SOCE in hepatocarcinoma cells by using siRNA approach. The successful knockdown of Orai1 was demonstrated by Western blotting (Fig. S3A). Down‐regulation of Orai1 led to a decrease in SOCE evoked by Tg in HepG2 cells without affecting ER Ca^2+^ release (Fig. 4E and F). To better understand the link between Orai1‐mediated SOCE and AKT/mTOR signalling, HepG2 cells were transfected with Orai1 siRNA in the presence or absence of Tg treatment. Western blotting showed that knockdown of Orai1 expression was associated with a decrease in AKT and mTOR phosphorylation. Furthermore, the activation of AKT/mTOR was also inhibited in Orai1 siRNA‐treated cells that were induced by Tg. Consequently, silencing Orai1 increased LC3B‐II formation and Beclin‐1 expression and restored Tg‐induced the decrease in LC3B‐II and Beclin‐1 expressions (Fig. 4G and H). These results indicate that Orai1 may play an important role in autophagy‐dependent PI3K/AKT/mTOR pathway.

### Inhibition of Orai1 or SOCE enhances 5‐FU‐induced autophagy and cell death

**Figure 5:**
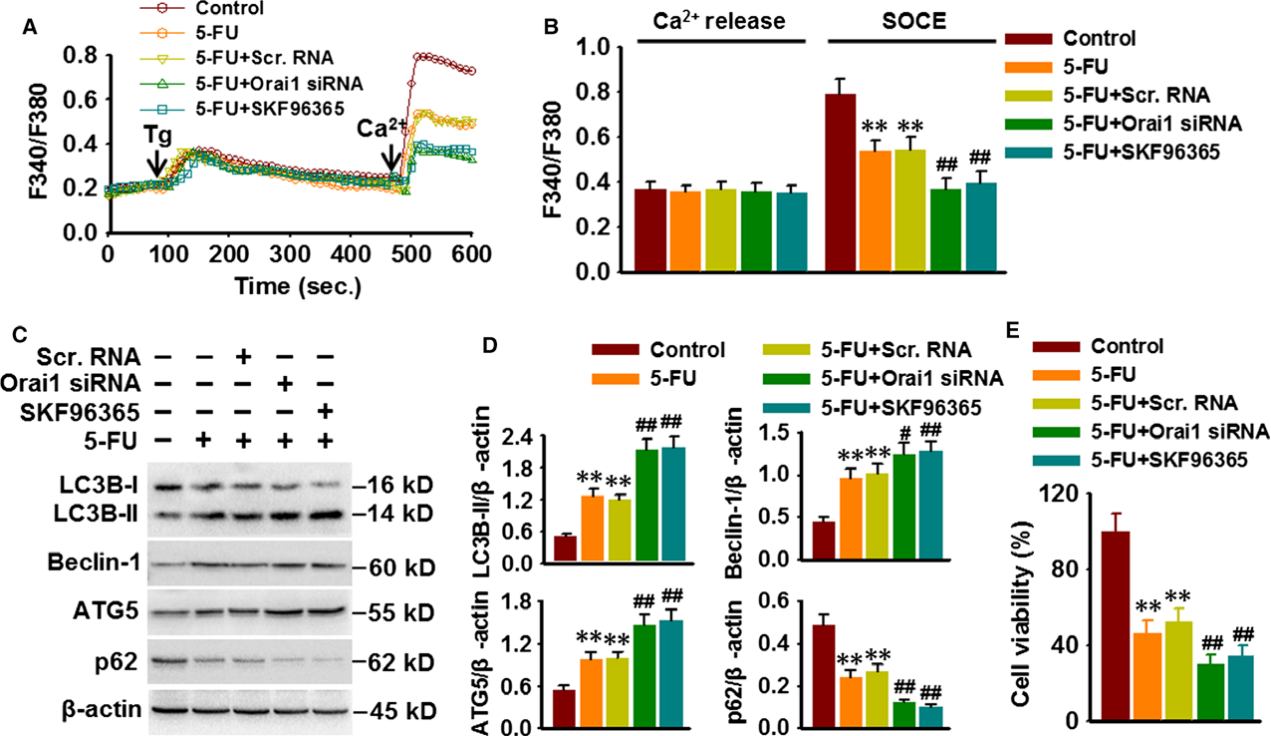
Inhibition of Orai1 or SOCE enhances 5‐FU‐induced autophagy and cell death. (**A**) Cells were treated with Orai1 siRNA (50 nM) for 48 hrs or treated with SKF96365 (20 μM) for 3 hrs prior to 5‐FU (80 μM) incubation. At the end of the experiment, cells were loaded with Fura2/AM probe and subjected to Ca^2+^ imaging experiment. (**B**) Quantification of fluorescence ratio (340/380). **P < 0.01 *versus* control, ##*P* < 0.01 *versus* 5‐FU,* n* = 6. (**C**) Representative Western blotting images showing the expression of LC3B, Beclin‐1, ATG5 and p62. (**D**) Densitometric analysis of LC3B‐II, Beclin‐1, ATG5 and p62 protein expression was performed. **P < 0.01 *versus* control, #*P* < 0.05, ##*P* < 0.01 *versus* 5‐FU,* n* = 5. (**E**) HepG2 cell viability was analysed by CCK‐8 assay. **P < 0.01 *versus* control, ##*P* < 0.01 *versus* 5‐FU,* n* = 4.

To clarify whether 5‐FU‐induced autophagic death is associated with Orai1‐mediated SOCE, Ca^2+^ entry and autophagy‐associated proteins expression were examined in HepG2 cells transfected with Orai1 siRNA or treated with SOCs inhibitor SKF96365 (20 μM) following 5‐FU challenge. The results demonstrated that knockdown of Orai1 expression and inhibition of SOCs both clearly retarded [Ca^2+^]_i_ increase induced by Tg (Fig. 5A and B). In addition, blockade of Orai1‐mediated Ca^2+^ entry further enhanced 5‐FU‐induced expression of LC3B‐II, Beclin‐1 and ATG5 and degradation of p62 (Fig. 5C and D). More importantly, the inhibitory effect of 5‐FU on cell viability was further augmented in HepG2 cells treated with Orai1 siRNA or SKF96365 (Fig. 5E). These data demonstrate that inhibition of Orai1‐mediated Ca^2+^ entry sensitizes HepG2 cells to 5‐FU chemotherapy treatment.

### Overexpression of Orai1 inhibits 5‐FU‐induced autophagic cell death

**Figure 6:**
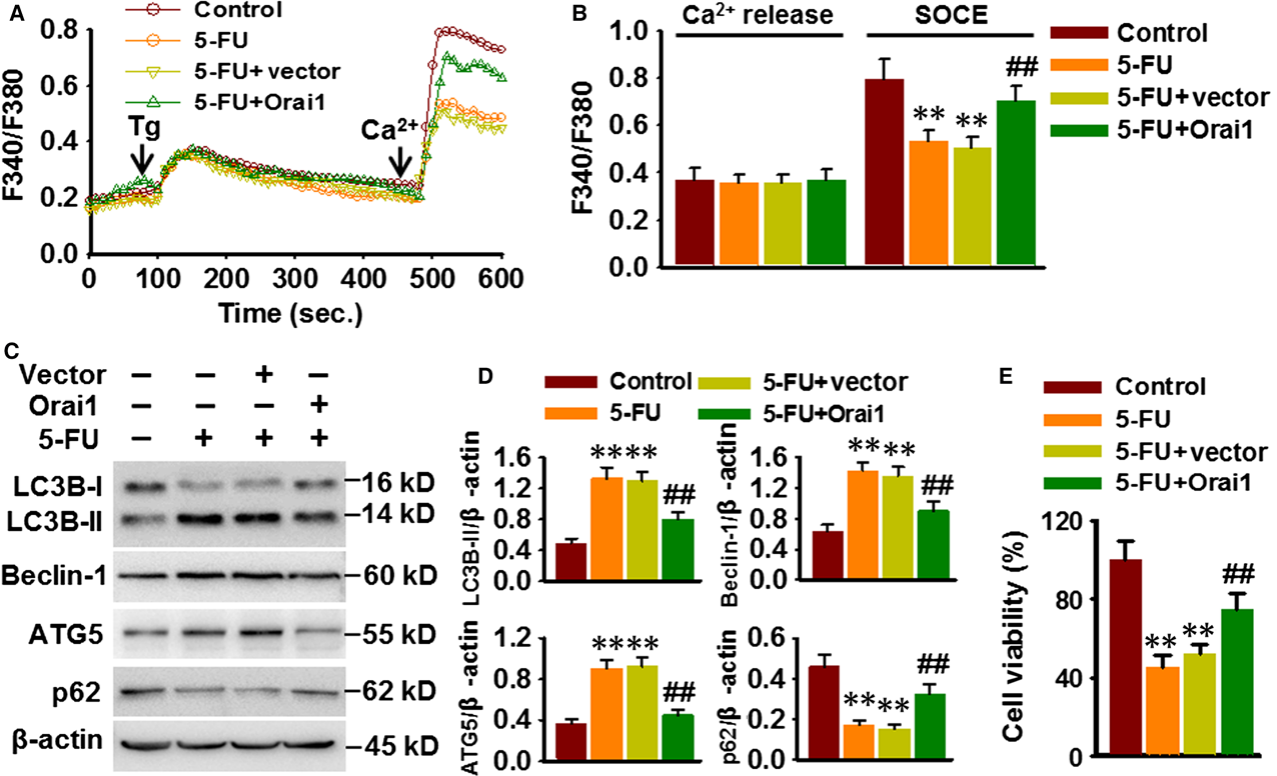
Restoration of Orai1 attenuates 5‐FU‐induced autophagic cell death. (**A**) Cells were transfected with Orai1 plasmid for 48 hrs prior to 5‐FU (80 μM) incubation for another 48 hrs. Ca^2+^ imaging experiment was performed as mentioned in methods section. (**B**) Quantification of fluorescence ratio (340/380). **P < 0.01 *versus* control, ##*P* < 0.01 *versus* 5‐FU,* n* = 6. (**C**) Representative Western blotting images showing the expression of LC3B, Beclin‐1, ATG5 and p62. (**D**) Densitometric analysis was performed using ImageJ analysis software. **P < 0.01 *versus* control, ##*P* < 0.01 *versus* 5‐FU,* n* = 5. (**E**) Cell viability was examined by CCK‐8 assay. **P < 0.01 *versus* control, ##*P* < 0.01 *versus* 5‐FU,* n* = 6.

To further confirm the role of Orai1‐mediated Ca^2+^ entry in regulating 5‐FU‐induced autophagic cell death, the expression of autophagy‐associated molecules and cell viability was determined in Orai1 overexpressed HepG2 cells following 5‐FU treatment. Transfection of Orai1 plasmid obviously increased Orai1 expression compared with vector or control group (Fig. S3B). Correspondingly, Orai1 overexpression blunted 5‐FU‐induced decrease in [Ca^2+^]_i_ following Tg challenge (Fig. 6A and B). Moreover, 5‐FU‐induced increase in LC3B‐II, Beclin‐1 and ATG5 expression and decrease in p62 expression were reversed in cells transfected with Orai1 plasmid (Fig. 6C and D). Furthermore, Orai1 overexpression significantly antagonized 5‐FU‐induced cell death (Fig. 6E). Together, these results indicate that Orai1 overexpression could attenuate the chemosensitivity of HepG2 cells to 5‐FU.

### Orai1 blocks 5‐FU‐induced autophagy through AKT/mTOR signalling pathway

**Figure 7:**
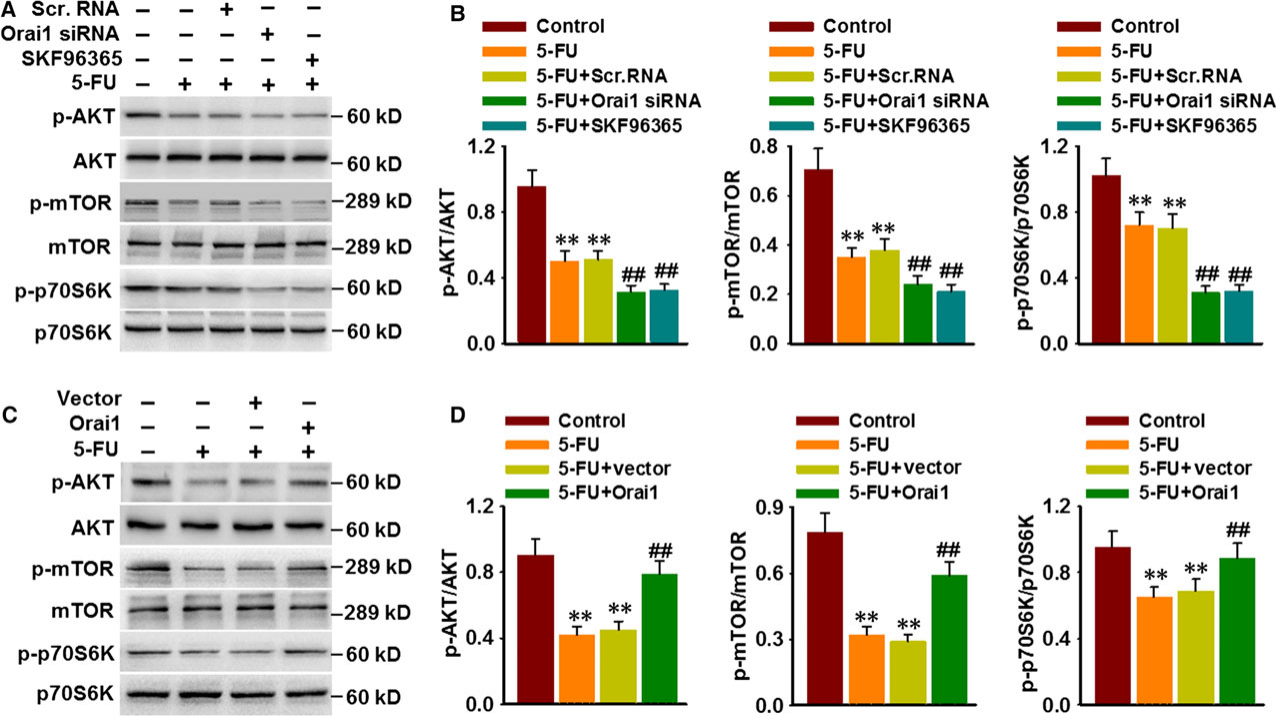
Orai1 inhibits 5‐FU‐induced autophagy through AKT/mTOR signalling pathway. (**A**) HepG2 cells were treated with Orai1 siRNA (50 nM) for 48 hrs or treated with SKF96365 (20 μM) for 3 hrs prior to 5‐FU (80 μM) incubation for another 48 hrs. The phosphorylation of AKT, mTOR and p70S6K were determined by Western blotting. (**B**) Bar diagram represents the densitometric analysis of the phosphorylation level of above proteins. **P < 0.01 *versus* control, ##*P* < 0.01 *versus* 5‐FU,* n* = 5. (**C**) Cells were transfected with Orai1 plasmid for 48 hrs and then were treated with 5‐FU (80 μM) for another 48 hrs. Representative Western blotting images showing the phosphorylation of AKT, mTOR and p70S6K. (**D**) Densitometric analysis of AKT, mTOR and p70S6K phosphorylation was performed. **P < 0.01 *versus* control, ##*P* < 0.01 *versus* 5‐FU, *n* = 6.

We have provided the evidence that PI3K/AKT/mTOR axis is an essential signalling pathway involved in 5‐FU‐induced autophagy. To unveil the mechanism through which Orai1‐mediated Ca^2+^ entry attenuates 5‐FU‐induced autophagic death, the phosphorylation of this signalling pathway was determined. The results in Figure 7A and B showed that 5‐FU‐induced decreases in AKT, mTOR and p70S6K phosphorylation were more obvious in HepG2 cells after Orai1 siRNA or SKF96365 treatment. In contrast, Orai1 overexpression remarkably blocked 5‐FU‐induced decrease in AKT, mTOR and p70S6K phosphorylation (Fig. 7C and D). These data suggest that Orai1‐mediated Ca^2+^ entry controls autophagy through regulating PI3K/AKT/mTOR signalling pathway.

## Discussion

In present study, we provide the evidence that 5‐FU can induce autophagic cell death in HepG2 hepatocarcinoma cells. 5‐FU activates autophagy through inhibition of PI3K/Akt/mTOR signalling pathway. In addition, 5‐FU decreases Orai1 expression and SOCE in HepG2 cells. Blockade of Orai1‐mediated Ca^2+^ entry potentiates 5‐FU‐induced autophagic cell death. In contrast, ectopic expression of Orai1 antagonizes 5‐FU‐activated autophagy. These findings demonstrate that Orai1‐mediated Ca^2+^ entry may be a critical determinant of 5‐FU‐induced autophagic cell death in HepG2 cells.

Induction of cell apoptosis has been proposed to be the major mechanism to explain the anticancer effect of 5‐FU. Interestingly, recent accumulating evidence has demonstrated that 5‐FU can induce autophagy in several cancer cells including colon 4, 25, pancreatic 15 and gastric cancer cells 17. Consistent with these results, our present work clearly showed that 5‐FU activates autophagy in HepG2 hepatocarcinoma cells basis on the observation that 5‐FU treatment increased expression of some key autophagy‐specific genes (Atg) including LC3B‐II, Beclin‐1 and ATG5. Particularly, 5‐FU increased punctate LC3B staining and promoted the conversion of LC3B‐I to LC3B‐II, the process reflecting the numbers of autophagosome formation. Moreover, we found that inhibition of autophagosomes degradation using chloroquine blocked 5‐FU‐induced decrease in p62 expression and further enhanced conversion of LC3B‐I to LC3B‐II in HepG2 cells, suggesting 5‐FU‐induced autophagy is ascribe to increases of autophagosomes formation rather than reduction in autophagosomes degradation.

Notably, the contribution of autophagy to 5‐FU‐induced cancer cell death is still controversial. Several reports indicated that autophagy acts as the predominant cell death mechanism in response to chemotherapy 13, 15, whereas others reported that autophagy serves as a pro‐survival signalling which could induce chemoresistance to anticancer drugs 4, 16, 25. For example, genistein potentiates 5‐FU‐induced autophagy in human pancreatic cancer cells, which ultimately leads to cell apoptosis 15. Contrarily, CD44v6 enhances 5‐FU‐induced autophagy flux and contributes to chemoresistance in SW480 cells under cytotoxic stress 25. Moreover, activation of JNK signalling confers 5‐FU resistance in colon cancer cells by promoting autophagy as a pro‐survival effect 4. Our data here revealed that inhibition of autophagy using 3‐MA suppressed the anticarcinogenic effect of 5‐FU, demonstrating autophagy is an important pathway involving in 5‐FU‐induced hepatocarcinoma cell death. The discrepancy could be probably due to multiple factors, such as cell type, pathology, the severity of the autophagy and intracellular signalling pathway involved 26, 27.

Ca^2+^, an important intracellular second messenger, plays a vital role in regulating various cellular functions such as cell proliferation, survival, differentiation and apoptosis 14, 28, 29. Recently, an increase in SOCE, related to the up‐regulation of Stim1, Orai1 or TRPC1 expressions, has been observed in several different kinds of tumours 6, 24, 30, indicating SOCs are the potential therapeutic target for treatment of cancers. In hepatocarcinoma, recent studies have shown that inhibition of TRPC1 inhibits cell proliferation by regulating SOCE 7, 12. Moreover, Stim1 expression is found to increase in hepatoma tissues than in precancerous tissues, and blockade of Stim1‐mediated SOCs inhibits migration and invasion of hepatocarcinoma cells 6. Our results in this study further showed another critical component of SOCs, Orai1 expression was increased in hepatocarcinoma tissues, indicating the changes of Orai1 expression may also serve as a casual factor in the development of hepatocarcinoma. More importantly, in line with the pro‐survival role of Stim1 6, ectopic expression of Orai1 in HepG2 cells blocked 5‐FU‐induced autophagic cell death, whereas knockdown of Orai1 or inhibition of SOCE potentiated 5‐FU‐induced autophagic cell death. These data indicate that Orai1‐mediated Ca^2+^ entry plays an essential role in regulating 5‐FU‐induced autophagic cell death in hepatocarcinoma cells. Indeed, 5‐FU treatment obviously decreased SOCE in HepG2 cells. Additionally, SOCE inhibition after 5‐FU treatment is due to the down‐regulation of Orai1, because 5‐FU significantly decreased Orai1 expression but had no effect on Stim1 phosphorylation, and Stim1 and TRPC1 expressions. These findings together suggest that Orai1 expression level may be a critical determinant of chemosensitivity of hepatocarcinoma cells to 5‐FU.

The critical role of [Ca^2+^]_i_ in regulating autophagy has been well documented in different types of cells 13, 14, 29, 31, 32. Activation of inositol 1,4,5, triphosphate (IP3) receptor (IP3R) could induce Ca^2+^ release from the Ca^2+^ store and evoke a transient increase of [Ca^2+^]_i_, leading to the inhibition of autophagy 31, 33. In prostate cancer cells, activation of SOCE reduces autophagosome formation, whereas inhibition of Stim1 expression and SOCE results in autophagic cell death 13. In present study, we provided convincing evidence that Orai1‐mediated Ca^2+^ entry blocked 5‐FU‐induced autophagic cell death in HepG2 cells *via* activation of PI3K/AKT/mTOR signalling pathway. Interestingly, we found that inhibition of PI3K class I enhanced autophagy, whereas class III attenuated 5‐FU‐induced autophagy, indicating 5‐FU induces autophagy *via* inhibition of class I PI3K/AKT/mTOR pathway. This is in accordance with the results of the recent studies that class III PI3K participates in autophagy initiation and progression and AKT/mTOR pathway is often activated by class I PI3K 34, 35. The latter is essential for inhibition of autophagy 13. Collectively, our data supported that [Ca^2+^]_i_ is a negative regulator of autophagy and prevents against chemotherapy‐induced cell death. Of note, however, the opposite results about the role of [Ca^2+^]_i_ in autophagy have also been reported in human salivary gland cells. Ca^2+^ entry *via* TRPC1 in these cells acts as an inducer of autophagy 29. The distinct role of Ca^2+^ influx *via* different SOCs in regulation of autophagy may be the possible explanation for the inconsistency. Another explanation for the discrepancy may come from different mechanisms of these SOCs involved in autophagy, because TRPC1 has been proposed to inhibit IP3R‐mediated anti‐autophagic Bcl‐2‐Beclin‐1 complex formation and thus induce autophagy through interaction with IP3R 29, 32.

In conclusion, our data in present study provided the strong evidence to show that Orai1 expression is increased in hepatocarcinoma tissues. 5‐FU can induce autophagic cell death in HepG2 hepatocarcinoma cells through inhibiting Ca^2+^ entry *via* down‐regulation of Orai1 expression. Ectopic expression of Orai1 prevents against 5‐FU‐induced cell death. These findings suggest that Orai1 expression may act as a predictor of 5‐FU sensitivity for hepatocarcinoma treatment. Blockade of Orai1‐mediated SOCE may be a potential strategy to sensitize hepatocarcinoma cells to 5‐FU treatment.

## Author contributions

R.P., S.L., J.Z. conceived and designed the experiments; B.T., X.X., X.L. performed the experiments; B.Y., J.Y., X.M., J.S. analysed the data; R.P., S.L. wrote the manuscript; J.Z. gave conceptual advice and edited the manuscript. All authors discussed the results and commented on the manuscript at all stages.

## Conflicts of interest

The authors confirm that there are no conflicts of interest.

## Supporting information


**Figure S1** 5‐FU treatment has no effects on Stim1 and TRPC1 expression, and Stim1 phosphorylation
**Figure S2** Orai1 protein expression in 15 pairs of liver cancer and adjacent normal tissues was analyzed by western blotting
**Figure S3** Effects of Orai1 siRNA or plasmid transfection on Orai1 protein expression
