# Supplementary material for: Inhibition of Orai1‐mediated Ca2+ entry enhances chemosensitivity of HepG2 hepatocarcinoma cells to 5‐fluorouracil
Source: J Cell Mol Med. 2016 Nov 23;21(5):904–15. doi: 10.1111/jcmm.13029 (PMC5387165; doi:10.1111/jcmm.13029)
Supplement: Supplementary file 1 — Figure S1 5‐FU treatment has no effects on Stim1 and TRPC1 expression, and Stim1 phosphorylation Figure S2 Orai1 protein expression in 15 pairs of liver cancer and adjacent normal tissues was analyzed by western blotting Figure S3 Effects of Orai1 siRNA or plasmid transfection on Orai1 protein expression [file JCMM-21-904-s001.doc]

**Inhibition of Orai1-mediated Ca^2+^ entry enhances chemosensitivity of HepG2** **hepatocarcinoma cells to 5-fluorouracil**

Bao-Dong Tang^1^, PhD^*^; Xin Xia^2^, MS^*^; Xiao-Fei Lv^3^, PhD^*^; Bei-Xin Yu^3^, PhD; Jia-Ni Yuan^3^, PhD; Xiao-Yi Mai^3^, PhD; Jin-Yan Shang^3^, PhD; Jia-Guo Zhou^3,4^, PhD; Si-Jia Liang^3^, PhD; Rui-Ping Pang^4,5^, PhD

^1^Department of Gastroenterology, the First Affiliated Hospital, ^2^Department of Thoracic Surgery, the First Affiliated Hospital, ^3^Department of Pharmacology, Cardiac and Cerebrovascular Research Center, Zhongshan School of Medicine, ^4^Guangdong Province Key Laboratory of Brain Function and Disease, Zhongshan School of Medicine, ^5^Department of Physiology, Pain Research Center, Zhongshan School of Medicine, Sun Yat-Sen University, Guangzhou, 510080, China

Running title: Orai1 and autophagic cell death

Correspondence to:

Dr. Rui-Ping Pang, Department of Physiology, Pain Research Center, Zhongshan School of Medicine, or Si-Jia Liang, Department of Pharmacology, Cardiac and Cerebrovascular Research Center, Zhongshan School of Medicine, Sun Yat-Sen University, 74 Zhongshan 2 Rd, Guangzhou, 510080, China.

Tel: 86-20-87331956, Fax: 86-20-87331956

E-mail: [pangruip@mail.sysu.edu.cn](mailto:pangruip@mail.sysu.edu.cn) or liangsj5@mail.sysu.edu.cn

*These authors contributed equally to this work.

**Supplementary Figures**

Figure S1


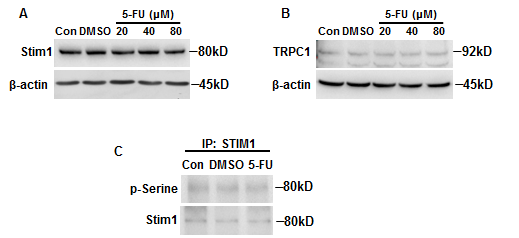


Figure S1. 5-FU treatment has no effects on Stim1 and TRPC1 expression, and Stim1 phosphorylation. (A and B) HepG2 cells were treated with different concentrations of 5-FU for 48 h, Stim1 (A) and TRPC1 (B) expression were determined by western blotting. (C) The serine phosphorylation of Stim1 was then examined by immunoprecipitation.

Figure S2


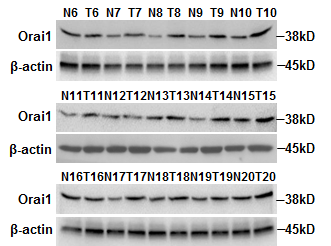


Figure S2. Orai1 protein expression in 15 pairs of liver cancer and adjacent normal tissues was analyzed by western blotting.

Figure S3


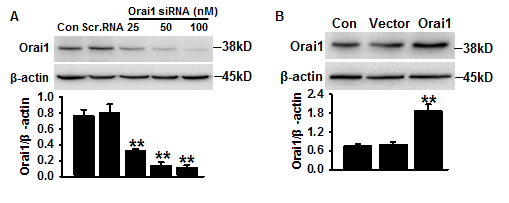


Figure S3. Effects of Orai1 siRNA or plasmid transfection on Orai1 protein expression. (A and B) Western blotting showing the expression of Orai1 in Hep2 cells transfected with Orai1 siRNA (A) or Orai1 plasmid (B) for 48 h. **p<0.01 vs. Scr.RNA or vector (pcDNA 3.1), n=4.
